# Editorial: Updates on the role of surfactant proteins A and D in innate immune responses

**DOI:** 10.3389/fimmu.2022.1113210

**Published:** 2022-12-13

**Authors:** Taruna Madan, Nicole M. Thielens

**Affiliations:** ^1^ Department of Innate Immunity, ICMR-National Institute for Research in Reproductive and Child Health, Mumbai, India; ^2^ Université Grenoble Alpes, CNRS, CEA, IBS, Grenoble, France

**Keywords:** SP-A, SP-D, alveolar macrophages, SNP, prostate cancer, SARS-CoV-2, RSV, lung diseases

Surfactant proteins A (SP-A) and D (SP-D) are collagen-containing calcium-dependent lectins, serving as soluble innate immune molecules. They maintain pulmonary and extrapulmonary homeostasis through their multiple roles as anti-infectious, immunomodulatory, and anti-cancer agents. These proteins bind to target ligands on pathogens, allergens, tumor and apoptotic cells, bringing about the effector functions *via* their interaction with cell surface receptors, enhancing target clearance from mucosal entry points and modulating the inflammatory response. Recombinant molecules of SP-A and SP-D may represent potential therapeutic strategies, with their actions aiding the blockade of viral, bacterial, and fungal infections and their immunomodulatory capacity to prevent excessive inflammatory responses. The eleven original research manuscripts of this themed collection highlight recent achievements regarding the role of SP-A and SP-D in innate immune responses.

Four of the articles relate to lung alveolar cells, including alveolar macrophages (AMs) which are the main cells involved in SP-A/D-mediated immune response in the lung. The first study by Wang et al. demonstrated that intratracheal spraying of engineered nanomaterials (ENMs) of four different types (fullerene, carbon nanotubes, titanium or silicon dioxides) induced inflammation in the rat lung, most of the administered ENMs being phagocytosed by AMs. *In vitro* analyses in the presence of rat bronchoalveolar lavage fluid (BALF) revealed that SP-A/D opsonized all tested ENMs and enhanced their phagocytosis by AMs through CD14 whereas cytokine production was dependent on the type of ENM phagocytosed. SP-A/D, therefore, play an important role by opsonizing inhaled nanoparticles, thereby contributing to decreased lung toxicity.

Two contributions from the group of J. Floros investigated changes in the toponome of AMs and in the miRNome of lung alveolar cells (LACs) of SP-A knockout (KO) mice following Klebsiella pneumoniae exposure. Phelps et al. employed the toponome imaging system (TIS) with serial immunostaining of cells for the localization of multiple markers, allowing characterization of multi-protein clusters, referred to as Combinatorial Molecular Phenotypes (CMPs). They reported a decrease of CMPs exclusive to infected AMs in KO mice, which may underlie the susceptibility of these mice to infection. In addition, both KO groups (infected and non-infected) exhibited more exclusive CMPs than the corresponding WT groups, suggesting that SP-A may participate directly to CMP formation through interactions with various cell surface molecules. As underlined by the authors, future studies will require exploration of CMP differences by using additional markers and/or by influencing specific markers by exogenous SP-A treatment. Thorenoor and Floros studied the role of exogenous SP-A rescue on the regulation of the LACs miRNome, the targets of miRNA-RNA, and the gene expression in SP-A-KO infected male and female mice. All analyses reveal sex differences and involvement of the miRNA-RNA targets in inflammation, anti-apoptosis and cell cycle pathways. The gene expression profiles identify the TNF, cell cycle, and TP-53 (known to play a role in cancer) signaling nodes under the studied conditions. These findings are relevant to be considered for the potential therapeutic use of SP-A in pulmonary diseases.

The fourth study by Garcia-Fojeda et al. focused on SP-A-dependent signaling pathways that enhanced IL-4 mediated activation of tissue repair program in AMs. They showed that SP-A activated PI3K-Akt dependent signaling pathways and contributed to increased proliferation and alternative activation of AMs. In addition, SP-A and IL-4 synergistically enhanced mitochondrial respiration and glycolysis in AMs. The signaling events that drive the SP-A-dependent amplification of IL-4 effects on AMs may aid in the development of new approaches to control lung diseases caused by exaggerated repair responses.

Understanding on the genetic basis of the role of SPs in lung diseases and infections has been enhanced by three studies. A contribution by Abbasi et al. compared single nucleotide polymorphisms (SNPs) of SP genes in two interstitial lung diseases, Idiopathic Pulmonary Fibrosis (IPF) and Hypersensitivity Pneumonitis (HP), in previously described Mexican study groups. Novel computational models were used to assess the epistatic effects of SNP-SNP interactions in both the diseases. The results show that IPF and HP are associated with some common SP genetic variants, suggesting shared pathophysiological mechanisms. However, one of the SNP interactions involving the *SFTPA1*, *SFTPA2* and *SFTPD* genes is associated with a disease-specific outcome, suggesting that specific interactions may serve as markers to distinguish between epithelial-driven fibrosis (IPF) and inflammatory-driven fibrosis (HP).

To understand if the genetic heterogeneity of SP-A is the reason for its dysfunctionality in asthma, Francisco et al., evaluated the functional significance of a single nucleotide polymorphism within the carbohydrate recognition domain of Surfactant Protein-A2 (SP-A2), which results in an amino acid substitution at position 223 from glutamine (Q) to lysine (K). With strong evidences of higher anti-asthmatic potential of SP-A 223Q in the asthmatics, humanized mice and the primary bronchial epithelial cells from asthmatics, the authors developed 10 and 20 amino acid peptides of SP-A2 spanning position 223Q and the exogenous administration of SP-A 223Q peptides significantly lowered eosinophilic inflammation, mucin production and airways hyperresponsiveness in a house dust mite model of asthma, protect from lung function decline during an IL-13 challenge model in mice, and decrease IL-13-induced MUC5AC gene expression in primary airway epithelial cells from asthmatics.

Depicolzuane et al., tested the hypothesis that RSV severity in infants is associated with single nucleotide polymorphisms (SNPs) of surfactant proteins (SPs) in a prospective cohort of 405 RSV-positive children classified into moderate and severe. Increased risk of severe disease was associated with rs1059047_C of the SFTPA1 whereas the rs17886395_C of the SFTPA2 and rs2243639_A of the SFTPD were associated with protection. Importantly, the risk to severity was not associated with SNPs of hydrophobic SFTPB and SFTPC.

Two studies investigated the role of SP-A and SP-D in host defense against SARS-CoV-2. Hsieh et al., provided *in vitro* evidences for the protective role of rfhSP-D against SARS-CoV-2 infection. With a dose-responsive binding to S1 spike protein of SARS-CoV-2 and its receptor binding domain, rfhSP-D significantly inhibited interaction of S1 protein with the hACE2 overexpressing HEK293T cells. A ~0.5 RLU fold reduction in the entry of pseudotyped lentiviral particles validated the therapeutic potential of rfhSP-D.

Aramyan et al., utilized a hybrid quantum and classical *in silico* modeling technique that utilized protein graph pruning and indicated that SP-A may ligate the S protein with a similar affinity to the ACE2-Spike binding. With the high affinity SP-A binding site localized to the fusion region of the SARS-CoV-2 S protein, responsible for viral entry, the authors speculate that SP-A may not directly compete with ACE2 for the binding site on the S protein, but may interfere with viral entry to the cell by hindering necessary conformational changes or the fusion process.

To explore a novel solution to the growing problem of multidrug-resistant bacteria, Coya et al., investigated the therapeutic impact of human SP-A and a recombinant trimeric fragment (rfhSP-A) together with antibiotics against pathogenic Gram-negative bacteria. SP-A directly interacted with polymyxin B and showed synergistic microbicidal activity with polymyxin B and E against three SP-A-resistant pathogenic bacteria: Klebsiella pneumoniae, non-typable Haemophilus influenzae (NTHi), and Pseudomonas aeruginosa facilitated by the formation of SP-A/PMB aggregates and enhanced bacterial membrane permeabilization. A molecular derivative of PMB lacking the acyl chain (PMBN) with binding ability but no bactericidal activity showed synergistic bactericidal activity against Gram-negative bacteria along with SP-A. The trimeric rfhSP-A fragment had relatively lower but significant direct bactericidal activity against *K. pneumoniae*, NTHi, and *P. aeruginosa*.

Novel anti-tumoral immune mechanisms of SP-D in prostate cancer (PCa) have been elucidated by Ganguly et al.. These are suggestive of transformation of an immunologically “cold tumour” into a “hot tumour”, thereby implicating possible synergistic potential of recombinant fragment of human SP-D (rfhSP-D) along with the immune check point inhibitor therapy. Decreased expression and increased proteolytic degradation of SP-D was observed in early and advanced stages of PCa in the transgenic adenocarcinoma of mouse prostate (TRAMP) model associated with increased serine proteases producing granulocytes and polymorphonuclear myeloid-derived suppressor cells (PMN MDSCs). Importantly, treatment of *ex-vivo* cultured TRAMP tumours with rfhSP-D and elastase inhibitor, Sivelestat led to polarization towards M1 macrophages with downregulation of PMN MDSCs and enhanced immunogenic cell death.

To summarise, this Research Topic provides much enhanced knowledge of the impact of SPs and their genetic heterogeneity on lung immunity and infections and on the immune component of prostate tumour microenvironment strengthening the premise of clinical application of SPs in various pulmonary diseases and cancer.

## Author contributions

Both TM and NMT have made a substantial, direct, and intellectual contribution to the Editorial and approved it for publication.

